# Genomic diversity of the pathogenic fungus *Aspergillus fumigatus* in Japan reveals the complex genomic basis of azole resistance

**DOI:** 10.1038/s42003-024-05902-6

**Published:** 2024-03-14

**Authors:** Xiaohui He, Yoko Kusuya, Daisuke Hagiwara, Takahito Toyotome, Teppei Arai, Cai Bian, Masaki Nagayama, Saho Shibata, Akira Watanabe, Hiroki Takahashi

**Affiliations:** 1 Medical Mycology Research Center, Chiba University, 1-8-1 Inohana, Chuo-ku, Chiba, 260-8673 Japan; 2 Biological Resource Center, National Institute of Technology and Evaluation, 2-5-8 Kazusakamatari, Kisarazu, 292-0818 Japan; 3 Faculty of Life and Environmental Sciences, University of Tsukuba, 1-1-1 Tennodai, Tsukuba, Ibaraki 305-8577 Japan; 4 Department of Veterinary Medicine, Obihiro University of Agriculture and Veterinary Medicine, Nishi 2-11, Inadacho, Obihiro, 080-8555 Japan; 5 BGI-Shenzhen, Yantian District, Shenzhen, 518083 China; 6 Graduate School of Medical and Pharmaceutical Sciences, Chiba University, 1-8-1 Inohana, Chuo-ku, Chiba, 260-8670 Japan; 7 Molecular Chirality Research Center, Chiba University, 1-33 Yayoi-cho, Inage-ku, Chiba, 263-8522 Japan; 8 Plant Molecular Science Center, Chiba University, 1-8-1 Inohana, Chuo-ku, Chiba, 260-8675 Japan

**Keywords:** Fungal genomics, Fungal ecology, Fungal infection, Fungal genetics, Fungal genetics

## Abstract

*Aspergillus fumigatus* is a pathogenic fungus with a global distribution. The emergence of azole-resistant *A. fumigatus* (AR*Af*) other than the TR-mutants is a problem in Japan. Additionally, the genetic diversity of *A. fumigatus* strains in Japan remains relatively unknown. Here we show the diversity in the *A. fumigatus* strains isolated in Japan as well as the complexity in the global distribution of the pathogenic strains. First, we analyzed the genome sequences of 171 strains from Japan as well as the antifungal susceptibility of these strains. Next, we conducted a population analysis of 876 strains by combining the available genomic data for strains isolated worldwide, which were grouped in six clusters. Finally, a genome-wide association study identified the genomic loci associated with AR*Af* strains, but not the TR-mutants. These results highlight the complexity of the genomic mechanism underlying the emergence of AR*Af* strains other than the TR-mutants.

## Abstract (web summary)

Whole-genome analysis of 876 *Aspergillus fumigatus* strains including 171 strains from Japan highlights the diversity in the *A. fumigatus* strains isolated in Japan as well as the complexity in the global distribution of the pathogenic strains.

## Introduction

The filamentous fungus *Aspergillus fumigatus*, which is distributed worldwide, is the most important pathogenic fungus among *Aspergillus* species associated with aspergillosis^1,2^. Azoles, such as voriconazole (VRCZ) and itraconazole (ITCZ), are the main antifungal compounds used to treat *A. fumigatus* infections^3^.

The number of azole-resistant *A. fumigatus* (AR*Af*) strains that have been identified has continued to increase over the past decade^4,5^, resulting in serious clinical implications^6^. It is widely accepted that the azole resistance of *A. fumigatus* was acquired through the use of medication (patient route) and the application of azole fungicides in the environment (environmental route)^7–11^. The mechanisms underlying the azole resistance of AR*Af* strains have been characterized on the basis of mutations in *cyp51A* (*erg11*), which encodes a 14-alpha sterol demethylase targeted by azole antifungal compounds. Specifically, several point mutations (i.e., G54, G138, P216, M220, and G448) may be associated with gene structural changes^7–9^. Moreover, a 34 bp tandem repeat (TR_34_) in the promoter region of *cyp51A* along with a nucleotide change that results in the substitution of leucine 98 to histidine (TR_34_/L98H) as well as TR_46_/Y121F/T289A lead to gene overexpression^12,13^. The TR-type mutants are prevalent in Europe and the US^14^. In a previous study, 6.7% of the strains from soil samples were identified as AR*Af* in the UK^15^. The genetic diversity of the TR-mutants is low. Additionally, they have been grouped in a single population because they propagate through asexual reproduction^5,16^. In contrast to the situation in Europe, only a few TR-type mutants have been isolated in clinical and environmental settings in Japan^17^. A growing concern in Japan is the spread of AR*Af* through floriculture products, including tulip bulbs imported from the Netherlands^18–20^.

Notably, 43% of AR*Af* strains lack mutations in *cyp51A*^21^. Similarly, the surveillance in Germany and the US reported 47.1% and 65% of resistant isolates harboring the wild type *cyp51A* without any mutation, respectively^22,23^. Hence, the resistance mechanisms that do not involve a mutated *cyp51A* are currently being characterized. For example, the high basal expression of *cdr1B*, which encodes an ABC transporter, and mutations in *hmg1*, which encodes a hydroxymethylglutaryl-CoA (HMG-CoA) reductase (rate-determining enzyme in ergosterol biosynthesis), contribute to azole resistance^10,24,25^.

To investigate the heterogeneity of *A. fumigatus* genomes and AR*Af* resistance mechanisms, population genomics and pan-genomic analyses of a subset of *A. fumigatus* isolates collected worldwide have been conducted. There are many reports describing *A. fumigatus* genomes, including 300^26^ and 260^27^ genomes from *A. fumigatus* strains collected worldwide, 76 genomes from *A. fumigatus* strains from Japan^28^, 179 genomes from *A. fumigatus* strains from the US^14^, and 218 genomes from *A. fumigatus* strains collected across the UK and Ireland^29^. Moreover, microbial genome-wide association studies (GWAS) have been performed to identify mutations, including non-*cyp51A* mutations associated with azole resistance^28–30^. Zhao et al. detected mutations related to ITCZ sensitivity and validated the function of the candidate gene^28^. Although 17 strains^31,32^ and 76 strains susceptible to azoles^28^ obtained across Japan have been analyzed, the available information regarding the genetic diversity of *A. fumigatus* strains from Japan remains limited.

In this study, to explore the emergence of AR*Af* strains in Japan, we analyzed 171 strains (160 clinical strains, 10 environmental strains, and 1 strain from an unknown source), including previously reported strains^8,10,25,28,31,33^. First, we assessed the antifungal susceptibility of these strains, which revealed 22 strains, including 11 newly analyzed strains, with minimum inhibitory concentration (MIC) values ≥ 2 µg/mL. Next, to clarify the genetic diversity of *A. fumigatus* strains from Japan, we conducted a population analysis and a phylogenetic analysis using the genome sequences of 876 strains from the UK and Ireland, the US, Germany, Canada, Spain^34^, and the Netherlands in addition to the 171 strains from Japan, including 92 newly sequenced strains. We identified six clusters in the *A. fumigatus* population, with almost all of the strains from Japan assigned to Clusters 1, 2, and 4. Furthermore, using 628 strains in these three clusters, we performed a GWAS and detected the genomic loci associated with the azole resistance of the AR*Af* strains other than the TR-mutants. Finally, a ridge regression analysis revealed the complexity of the genomic mechanism underlying the emergence of AR*Af*. This study has elucidated the development of AR*Af* strains other than the TR-mutants, while also clarifying the genomic diversity of *A. fumigatus* strains from Japan.

## Results

### Characterization of AR*Af* strains

**Fig. 1:**
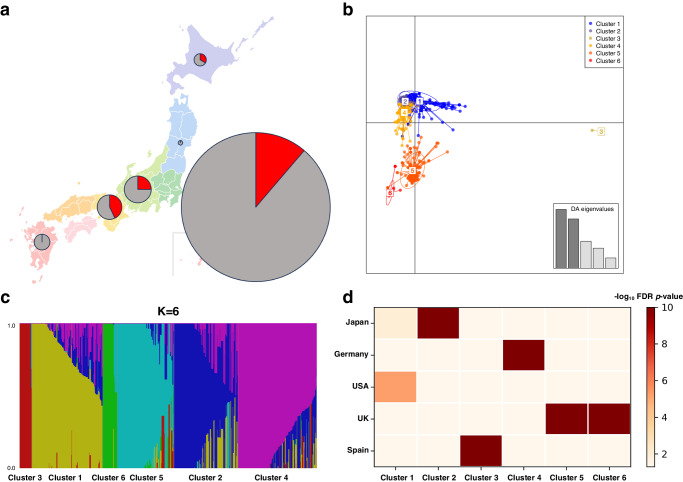
Population analyses of 876 genomes of *A. fumigatus*. **a** Map showing the strains from Japan included in this study. A total of 165 strains, not including six strains isolated from an unknown region, were mapped. More specifically, 142 strains (16 resistant strains) were from the Kanto region (Chiba, Ibaraki, and Tokyo), 8 strains (2 resistant strains) were from the Chubu region (Aichi, Gifu, Ishikawa, and Nagano), 7 strains (3 resistant strains) were from the Kinki region (Kyoto and Osaka), 4 strains were from the Kyushu region (Kagoshima, Kumamoto, and Fukuoka), 3 strains (1 resistant strain) were from the Hokkaido region, and 1 strain was from the Tohoku region (Iwate). Resistant and susceptible strains are indicated in red and gray, respectively. **b** DAPC scatterplot of the 876 strains. The optimal number of principal components (PC = 5) was estimated using the optim.a.score function implemented in the adegenet package. **c** Population structure plot (K = 6). The fastStructure analysis determined K = 6. **d** Overrepresentation of geographic distributions of the strains for each cluster.

A total of 173 strains were used, including 171 strains from Japan^8,10,25,28,31,33^ and the laboratory strains Af293^35^ and Afs35^36^. Most strains (83%; 134 clinical strains and 8 environmental strains) were isolated from the Kanto region (Chiba, Ibaraki, and Tokyo) in Japan (Fig. 1a). We determined the susceptibility of the strains to ITCZ on the basis of our analysis as well as the results of earlier studies (Supplementary Data 1). Twenty-two strains (13%) (21 clinical strains and 1 environmental strain) had MIC values ≥ 2 µg/mL. Accordingly, they were designated as AR*Af* (Table 1). Eleven of these 22 strains had not previously been identified as AR*Af*. To investigate the ITCZ resistance mechanisms, we confirmed the sequences of the *cyp51A* and *hmg1* alleles. Twenty strains, including serially isolated strains from seven patients (patient I, IFM 57543-2 and IFM 59984-1; patient II, IFM 60237 and IFM 65468; patient III, IFM 62103 and IFM 62105-1; patient IV, IFM 63240, IFM 63537-2, IFM 63714-1, and IFM 64173; patient V, IFM 63768 and IFM 63772; patient VI, IFM 64258, IFM 63805, and IFM 64259-1; and patient VII, IFM 63559-1 and IFM 63560-1), had mutations in *cyp51A* (G54R, G54W, G138C, H147Y, P216L, M220K, and G448S) and/or *hmg1* (S269F, S269Y, G307D, and F390Y). All identified variants were consistent with known alleles, indicating these mutations may be associated with azole resistance. Although IFM 62103 and IFM 62105-1 were isolated from the same patient (patient III), their mutation profiles differed. Two strains (IFM 62628 and IFM 63772) lacked mutated *cyp51A* and *hmg1* genes. Notably, the MIC values of IFM 63537-2 and IFM 63537 were 2 and 8 µg/mL, respectively^10^. Moreover, IFM 63537-2 was re-isolated from IFM 63537 via single colony isolation.

**Table 1:** Characteristics of the 22 AR*Af* strains in this study

Strain ID	Country, prefecture	Year of isolation	Source	MIC (μg/mL) ITCZ	Amino acid substitution		Reference
					*cyp51A*	*hmg1*	
IFM 57543-2^a^	Japan, Chiba	2007	Sputum (patient I)	2	G54R		This study
IFM 59984-1^a^	Japan, Chiba	2010	Sputum (patient I)	2	G54R		This study
IFM 60237^b^	Japan, Chiba	2011	Sputum (patient II)	4	P216L		Hagiwara et al.^8^
IFM 65468^b^	Japan, Chiba	2018	Sputum (patient II)	>8	P216L		This study
IFM 61578-1	Japan, Osaka	2012	BALF	4	P216L		Takahashi-Nakaguchi et al.^31^
IFM 62103^c^	Japan, Osaka	2012	Sputum (patient III)	2	H147Y		Arai et al.^25^
IFM 62105-1^c^	Japan, Osaka	2013	Sputum (patient III)	>8	M220K	G307D	Arai et al.^25^
IFM 62628	Japan, Chiba	2014	Soil	2			This study
IFM 63240^d^	Japan, Chiba	2014	Sputum (patient IV)	>8		S269F	Hagiwara et al.^10^; Arai et al.^25^
IFM 63537-2^d^	Japan, Chiba	2015	Sputum (patient IV)	2		S269F	Hagiwara et al.^10^
IFM 63714-1^d^	Japan, Chiba	2015	Sputum (patient IV)	>8		S269F	Hagiwara et al.^10^
IFM 64173^d^	Japan, Chiba	2016	Sputum (patient IV)	>8		S269F	Hagiwara et al.^10^
IFM 63768^e^	Japan, Chiba	2016	Sputum (patient V)	8		S269Y	Hagiwara et al.^10^
IFM 63772^e^	Japan, Chiba	2016	Sputum (patient V)	2			This study
IFM 64258^f^	Japan, Ibaraki	2016	Sputum (patient VI)	4		F390Y	Hagiwara et al.^10^
IFM 63805^f^	Japan, Ibaraki	2016	Sputum (patient VI)	>8	G138C		This study
IFM 64259-1^f^	Japan, Ibaraki	2016	Sputum (patient VI)	2	G138C		This study
IFM 63345	Japan, Tokyo	2014	Lung	>8	G54W		This study
IFM 63559-1^g^	Japan, Gifu	2014	Sputum (patient VII)	2	G448S		This study
IFM 63560-1^g^	Japan, Gifu	2015	Sputum (patient VII)	8	G448S		This study
IFM 64160-1 (OKH34)	Japan, Hokkaido	2015	Sputum	4	G448S		Toyotome et al.^33^
IFM 65494	Japan, Tokyo	2017	Sputum	8	P216L		This study

^a^The strains were serially isolated from patient I.

^b^The strains were serially isolated from patient II.

^c^The strains were serially isolated from patient III.

^d^The strains were serially isolated from patient IV.

^e^The strains were serially isolated from patient V.

^f^The strains were serially isolated from patient VI.

^g^The strains were serially isolated from patient VII.

Among the strains from a single patient, IFM 62103 and IFM 62105-1 from patient III harbored different mutations in *cyp51A* and *hmg1*, even though the short tandem repeat patterns were the same^25^. We identified 377 mutations between IFM 62103 and IFM 62105-1 (Supplementary Data 2). In addition to the missense variants in *cyp51A* and *hmg1*, 99 other missense variants were detected.

### Population structure of *A. fumigatus* strains in Japan

To clarify the population structure of the 171 strains from Japan, we analyzed 876 strains (31%; 183 resistant strains), including the 171 strains from Japan, 2 laboratory strains (Af293 and Afs35), 8 strains isolated from a single tulip bulb in Japan^18,20^, 212 strains from the UK and Ireland^29^, 12 strains from the Netherlands^37–39^, 256 strains from Germany^26^, 27 strains from Spain^34^, 10 strains from Canada^30^, and 178 strains (excluding AFIS1704) from the US^14^. We did not include AFIS1704 because its estimated genome size (64 Mb) differed considerably from the genome size (29 Mb) of Af293 (Supplementary Fig. 1).

Using 68,816 loci, we estimated the optimal number of populations on the basis of the discriminant analysis of principal components (DAPC). According to the Bayesian information criterion (BIC) with five principal components retained, K = 6 was the most likely number of populations (Fig. 1b, Supplementary Fig. 2 and Supplementary Data 3). In addition, fastStructure was used to estimate the number of populations. Because the marginal likelihood values increased until K = 6 (Supplementary Fig. 3), six clusters were supported by fastStructure (Fig. 1c and Supplementary Data 4). Cluster 4 was the largest with 241 strains (13%; 31 AR*Af* strains), followed by Cluster 1 with 214 strains (21%; 44 AR*Af* strains), Cluster 2 with 185 strains (15%; 27 AR*Af* strains), Cluster 5 with 175 strains (45%; 78 AR*Af* strains), Cluster 6 with 36 strains (89%; 32 AR*Af* strains), and Cluster 3 with 25 strains (8%; 2 AR*Af* strains) (Supplementary Data 5). Among the 171 strains from Japan, 58, 73, 33, and 7 strains were assigned to Clusters 1, 2, 4 and 5, respectively (i.e., no strains assigned to Clusters 3 and 6).

We assessed the geographic distributions of six clusters by Fisher’s exact test (Fig. 1d, Supplementary Fig. 4 and Supplementary Table 1). The six clusters were characterized by particular geographic regions. Clusters 1, 2, 3, and 4 were significantly overrepresented for the strains from the US (FDR corrected *p* = 5.85 × 10^−6^), Japan (FDR corrected *p* = 4.52 × 10^−11^), Spain (FDR corrected *p* = 2.18 × 10^−12^), Germany (FDR corrected *p* = 2.96 × 10^−12^), respectively. Clusters 5 and 6 were significantly overrepresented for the strains from the UK and Ireland (FDR corrected *p* = 7.16 × 10^−15^, *p* = 6.60 × 10^−15^).

The profiles of Tajima’s *D* values varied among Clusters 1, 2, 3, 4, 5 and 6 (Supplementary Fig. 5). The average Tajima’s *D* values for Clusters 1, 2, 3, 4, 5, and 6 were 0.65, 0.49, −0.56, 0.81, 0.99, and −1.31, respectively (Supplementary Table 2). The signature of positive selection was highest and lowest for Clusters 5 and 6, respectively (Supplementary Fig. 5), indicating the populations in Clusters 5 and 6 comprising TR-mutants may be under high evolutionary pressure, which is consistent with the use of azole fungicides in the field. Interestingly, the average Tajima’s *D* values and signature of positive selection differed among the chromosomes. More specifically, among the six clusters, Tajima’s *D* value for chromosome 6 was highest in Cluster 4, whereas Tajima’s *D* value for chromosome 8 was highest in Cluster 1, suggestive of the six cluster membership of the *A. fumigatus* population.

The comparison of the DAPC and fastStructure results revealed the cluster assignments were generally consistent. 26 strains were the exceptions (Supplementary Data 3 and 4). The differences between the strains in the cluster were evaluated by the numbers of pairwise single nucleotide polymorphisms (SNPs). Clusters 3 and 6 exhibited lower diversities (Supplementary Fig. 6 and Supplementary Table 3). Furthermore, we calculated *D*-statistics to test the admixture based on four clusters. Among 45 four-cluster comparisons, 9 and 20 comparisons exhibited significant *D*-statistics with Z-score > 3 and Z-score < −3, respectively, indicating that the admixture between most of clusters (Supplementary Fig. 7 and Supplementary Table 4).

To compare the recombination hot spots of clusters, we estimated the recombination rates for each cluster using LDhat analysis. The recombination rates for Clusters 1, 2, 3, 4, 5, and 6 were 0.2036/bp^−1^, 0.1215/bp^−1^, 0.0228/bp^−1^, 0.1320/bp^−1^, 0.1568/bp^−1^, 0.0078/bp^−1^, respectively. Cluster 1 exhibited the largest recombination rate. The greater numbers of recombination hot spots of Clusters 1, 2, 4 and 5 were detected than those of Clusters 3 and 6 (Supplementary Fig. 8). This is consistent with the numbers of pairwise SNPs (Supplementary Fig. 6), indicating that Clusters 3 and 6 could be highly clonal.

### Phylogenetic analysis

**Fig. 2:**
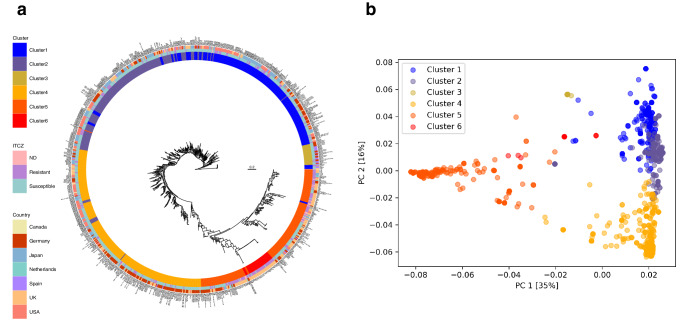
Phylogenetic analyses of 876 genomes of *A. fumigatus*. **a** Phylogenetic tree of 876 strains. RAxML was used to construct the unrooted phylogenetic tree. The metadata rings on the outside of the tree indicate the cluster, ITCZ resistance, and county. **b** Principal component analysis of 876 strains. The x-axis and y-axis correspond to principal components (PCs) 1 and 2, respectively; PC1 and PC2 explained 35% and 16% of the variance, respectively.

We conducted a phylogenetic analysis using the maximum likelihood method (Fig. 2a and Supplementary Data 5). By mapping six clusters on the phylogenetic tree, almost all strains were consistently assigned to their corresponding cluster. According to DAPC, 29 strains had a posterior probability of cluster membership <85%, indicating these strains may have been derived from the admixture between strains in two or more of the clusters.

On the basis of the principal component analysis (PCA), two populations (A and B) designated by Sewell et al^5^. were observed along with the first principal component (40% variation) (Fig. 2b). Indeed, Clusters 1, 2, and 4 were observed along with the second principal component (16% variation), while Clusters 5 and 6 were observed along with the first principal component (35% variation). The clusters defined by DAPC were the subclusters of populations A and B. Clusters 1, 2, and 4 were the subclusters of population B, whereas Clusters 5 and 6 were the subclusters of population A. Among the 131 TR-type mutants, 119 strains (93%) were assigned to Clusters 5 and 6. In contrast, the other TR-mutants were assigned to Clusters 1 (C87 and C91 from the UK and Ireland, and 698-L-3-11-2 from Germany), 2 (B11982, B11978, B11957, B11943, B11930, and B11927 from the US) and 4 (AB01_C43_NRZ-2018-313, AB01_C40_NRZ-2018-290, and AB01_C19_NRZ-2017-214 from Germany), but were positioned between populations A and B. The TR-mutants B11927, B11930, B11943, B11957, B11978, and B11982 had a 46% probability of belonging to Cluster 5 according to fastStructure.

### Genome-wide association study of the ITCZ resistance of *A. fumigatus*

**Fig. 3:**
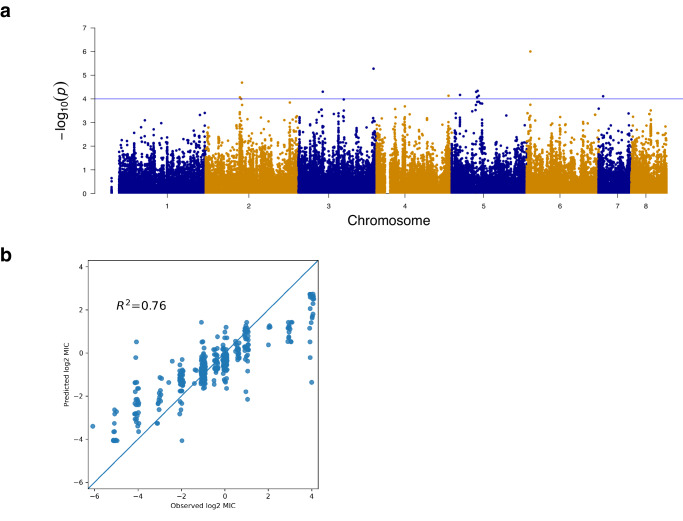
Genome-wide association study of the ITCZ resistance of *A. fumigatus*. **a** Manhattan plot. The azole resistance of AR*Af* was treated as a binary trait. The x-axis presents 90,648 loci, whereas the y-axis presents the *p*-values determined by TASSEL 5. **b** Fitting by ridge regression. The MIC value was treated as a quantitative trait. The x-axis presents log_2_-transformed MIC values, whereas the y-axis presents the predicted log_2_-transformed MIC values.

The AR*Af* strains other than the TR-mutants were mainly obtained in clinical settings in Japan. These strains were assigned to Clusters 1, 2, and 4 of population B. To explore the genomic loci of the AR*Af* strains other than the TR-mutants (i.e., high-risk population), we performed a GWAS involving 628 strains from Clusters 1, 2, and 4, of which 165 strains were from Japan. Among these 628 strains, 92 were AR*Af* strains, including 22 strains from Japan (Table 1), 22 strains from the UK and Ireland, and 14 strains from Germany (excluding the TR-mutants 698-L-3-11-2, AB01_C43_NRZ-2018-313, AB01_C40_NRZ-2018-290, and AB01_C19_NRZ-2017-214), 2 strains from Spain, 32 strains from the US (excluding the TR-mutants B11927, B11930, B11943, B11957, B11978, and B11982). Moreover, 46 strains had mutations in *cyp51A*, whereas the other 46 strains (50%) had no mutations in *cyp51A*. Only six strains had mutations in *hmg1*. The mixed linear model (MLM) analysis was conducted using TASSEL 5 (Fig. 3a and Supplementary Fig. 9). The azole resistance of the AR*Af* strains was treated as a binary trait. A total of 90,648 loci were filtered by allowing 10% missing values. The following 12 SNPs were significantly (*p* < 10^−4^) associated with AR*Af*: 1 missense variant, 1 synonymous variant, 1 intron variant and 9 intergenic variants (Table 2). Because 47 AR*Af* strains from the UK and Ireland, the US, Germany and Spain had no mutations in *cyp51A*, we screened for mutations in *hmg1*. We found seven strains with the mutations in *hmg1*, that is, E105K in C162, P309Q in C165 and CM7510, I419N in 106-C-1-72s-2, AB01_C6_NRZ-2016-108, and 313-H-1-15-2, and S541G in C4.

**Table 2:** Twelve significant SNPs associated with ITCZ resistance in Clusters 1, 2, and 4

Chr	Pos	Ref	Alt	*p*-value	Gene ID	Description	Variant
6	212,327	C	T	9.90 × 10^−7^			intergenic region
3	3,914,270	A	G	5.28 × 10^−6^	Afu3g14750	Fungal specific transcription factor, putative	intron region
2	1,896,813	G	A	2.05 × 10^−5^			intergenic region
5	1,372,777	G	A	4.58 × 10^−5^			intergenic region
3	1,266,532	A	G	4.97 × 10^−5^			intergenic region
5	1,295,108	G	A	5.04 × 10^−5^	Afu5g05550	Class V myosin	synonymous variant
5	453,949	C	T	6.81 × 10^−5^	Afu5g01780	Ortholog(s) have role in ascospore formation, conidium formation, hyphal growth, regulation of ascospore formation, regulation of meiosis I and regulation of mitotic nuclear division, more	missense variant (A329T)
4	3,753,479	T	C	7.36 × 10^−5^			intergenic region
5	1,430,964	G	A	7.38 × 10^−5^			intergenic region
7	239,634	C	T	7.74 × 10^−5^			intergenic region
5	1,344,332	G	A	8.49 × 10^−5^			intergenic region
2	1,787,428	T	C	8.55 × 10^−5^			intergenic region

The PCA of 628 strains revealed the overlap between the populations of AR*Af* and susceptible strains (Supplementary Fig. 10). This was consistent with the lack of strong signals detected by GWAS (Fig. 3a). Because GWAS assumes the phenotype can be explained by particular loci, we performed a genomic selection analysis via ridge regression to evaluate the effectiveness of each locus. The MIC values predicted by the ridge regression model were consistent with the observed MIC values (*R*^2^ = 0.76) (Fig. 3b), indicating that the MIC values could be explained by genomic loci. In accordance with the GWAS results, the coefficients of genomic loci determined by the ridge regression model contributed evenly to the MIC values (i.e., ITCZ resistance) (Supplementary Fig. 11). Considered together, these results indicate the azole resistance of the AR*Af* strains in Clusters 1, 2, and 4 may be associated with multiple loci, which is in contrast to the TR-mutants with a mutated *cyp51A* allele (L98H).

## Discussion

In this study, we analyzed 171 *A. fumigatus* strains isolated in Japan in terms of their susceptibility to ITCZ and their genomic sequences. By incorporating publicly available sequence data, we conducted a population analysis for the largest dataset, which resulted in the identification of six clusters. Furthermore, we conducted a GWAS to explore the genomic loci related to the azole resistance of the AR*Af* strains.

Among the 171 strains from Japan, 22 (13%) were AR*Af* strains, including non-*cyp51A* AR*Af* strains. In addition, 11 were newly identified, whereas 11 were previously reported strains (Table 1). Moreover, 14 strains (64%) harbored mutations in *cyp51A*. In contrast, seven strains (32%) harbored mutations in *hmg1*. Both IFM 62628 and IFM 63772 lacked mutations in these two genes. Interestingly, although the AR*Af* strains IFM 62103 and IFM 62105-1 were isolated from patient III and were clustered together (i.e., relatively close phylogenetic relationship), they differed regarding the *cyp51A* and *hmg1* mutations (Table 1 and Fig. 2a). Strain IFM 62103 harbored mutations in *cyp51A* (H147Y), whereas IFM 62105-1 harbored mutations in both *cyp51A* (M220K) and *hmg1* (G307D). Among the tested strains, IFM 62105-1 is reportedly the most resistant to ITCZ, VRCZ, and posaconazole^25^. By comparing the IFM 62103 and IFM 62105-1 genomes, we detected 377 variants, including 102 missense variants (Supplementary Data 2), suggesting that these strains may have other phenotypic differences in addition to the diversity in their susceptibility to azoles. Considering the similarity in the duration of azole treatments^25^, the mutations in different strains likely vary. Thus, various strains, including different AR*Af* strains, may have co-infected the lungs of patient III (i.e., heterogeneous population). Because heterogeneity may be advantageous for survival, heterogeneous populations are likely to emerge in human lungs. In addition, we detected a missense variant (I433N) in *clcA*, which encodes a Zn_2_-Cys_6_ transcription factor influencing hyphal growth, conidiogenesis, and adaptation to copper stress^40^. Mutations in *clcA* have been identified in both laboratory-based evolutionary analyses as well as in clinical settings^8,40^, suggesting that environmental stresses may induce *clcA* mutations that lead to adaptive responses.

We used 876 genomes, including the genomes of 854 strains with ITCZ susceptibility (MIC values or binary traits), to investigate the population structure of the strains from Japan (Supplementary Data 5). By analyzing the genomic context, we determined that the *A. fumigatus* strains can be divided into six clusters (Fig. 1b, c). The DAPC and fastStructure analyses supported the classification of the *A. fumigatus* strains in six clusters. The two *A. fumigatus* populations proposed by Sewell et al.^5^ were population A, which consists of TR-mutants, and population B, which typically does not include TR-mutants. For 212 strains by Rhodes et al.^29^, 116 (97%) and 89 strains (96%) were populations A and B, respectively. For 178 strains Etienne et al.^14^, 160 (100%) and 12 strains (67%) were populations A and B, respectively. The classification of two populations were consistent. In the current study, 165 strains from Japan were assigned to Clusters 1, 2, and 4 (i.e., subclusters of population B). Seven strains were assigned to Cluster 5 (i.e., subcluster of population A), but these strains were not TR-mutants. The population B could be abundant in Japan. In earlier studies, the number of populations ranged from two to seven^26–29,41^. We determined that PC1 explained 35% of the variation (Fig. 2b), which is less than the value (62%) reported by Etienne et al.^14^. This implies the subclusters were likely correct because expanding a strain set, especially Clusters 1, 2, and 4, may improve the resolution of the population structure. The optimal K value (i.e., 4) calculated by Zhao et al.^28^ for the strains from Japan was consistent with the results of the current study. In accordance with Clade 3 by Lofgren et al.^27^, Cluster 3 comprising of the strains from Germany and Spain was far from other clusters (Fig. 1b, c). The recombination analysis and Tajima’s *D* values indicated the high clonality of strains in Cluster 3 (Supplementary Figs. 5 and 8). Since the strains from Spain harbored unique *cyp51A*-3SNPs^42^, the mechanisms of AR*Af* strains belonging to Cluster 3 could be different. In addition, among the 53 non*-cyp51A* AR*Af* strains, 13 had mutations in *hmg1*, reflecting the importance of analyzing the *hmg1* allele as well as *cyp51A*. Especially, P309Q and I419N in *hmg1* are located in PF12349 (i.e., “sterol-sensing domain of SREBP cleavage-activation”), similar to S269F, suggesting that these alleles may be associated with azole resistance. Because the azole resistance mechanisms of the AR*Af* lacking *cyp51A* mutations remain unexplained, additional studies are required. Notably, the laboratory strains Af293 and Afs35 were assigned to different clusters, namely Cluster 1 of population B and Cluster 5 of population A, respectively (Fig. 1b), suggesting that these two laboratory strains may be useful for future research (depending on the study objectives). We excluded AFIS1704 from the population study because of the substantial difference in its estimated genome size (approximately 64 Mb) (Supplementary Fig. 1). Indeed, we confirmed the presence of two *mat1-2* and *cyp51A* genes on different contigs, suggesting that AFIS1704 may be an allodiploid hybrid strain (e.g., *Aspergillus latus*)^43^.

Strains from Clusters 1, 2, and 4 were included in the GWAS performed to explore the genomic loci related to azole resistance because they represent a high-risk population for the emergence of AR*Af* strains with mutations in *cyp51A* and/or *hmg1* (but are not TR-mutant strains). We identified 12 significant SNPs (*p* < 10^−4^), but there were no strong signals. These candidate SNPs were not overlapped with previous studies. Possibly, differently from GWAS for TR-mutants^29^ and azole resistance for all populations^30^, the GWAS for particular populations (Clusters 1, 2 and 4) could propose the novel SNPs in AR*Af*. In addition, we detected an overlap between the *cyp51A* AR*Af* strains and the susceptible strains in Clusters 1, 2, and 4 (Supplementary Fig. 10). Finally, a ridge regression analysis was conducted. The regression model explained the MIC values (Fig. 3b), but no significant loci were detected, consistent with the GWAS results. These findings suggest the phenotype of AR*Af* strains may be explained by multiple loci. The emergence of AR*Af* strains during azole treatments may occur randomly, regardless of the genomic background. In the GWAS analysis, clinical strains were overrepresented among 628 strains (Fisher’s exact test; *p*-value = 2.2 × 10^−8^). Since the genomic and metabolic differences between clinical and environmental strains have been reported^26,44^, the GWAS results might be potentially missing the aspects of environmental strains.

The results of this study revealed the diversity in the *A. fumigatus* strains isolated in Japan as well as the complexity in the global distribution of the pathogenic strains by using the largest dataset. Moreover, our findings complement the results of a previous study on the population structure of the isolates from Japan by Zhao et al.^28^. Furthermore, we identified significant loci related to AR*Af* strains, but not to TR-mutants. These candidate loci and their sequence data are relevant for future investigations conducted to conclusively determine how AR*Af* strains emerge in patients treated with azole-based antifungal compounds.

## Methods

### Strains and culture conditions

The strains used in this study (Supplementary Data 1) were isolated from various patients and environments in Japan from 1987 to 2018. All of the strains (IFM strains) are stored and maintained at the Medical Mycology Research Center, Chiba University in Japan. To prepare fresh conidia, the strains were grown on potato dextrose agar (BD Difco, Franklin Lakes, NJ) for 5–7 days at 37 °C.

### Antifungal susceptibility analysis

Antifungal susceptibility analyses were conducted using ITCZ in RPMI 1640 medium (pH 7.0) at 35 °C according to the Clinical and Laboratory Standards Institute reference broth microdilution method (document M38; 3rd edition)^45,46^ with minor modifications. Specifically, dried plates were used for evaluating antifungal susceptibility (Eiken Chemicals, Tokyo, Japan). The strains with a MIC value ≥ 2 µg/mL were defined as AR*Af*.

### Sequencing *cyp51A* and *hmg1* genes

The mutations in the *cyp51A* and *hmg1* genes were analyzed on the basis of a PCR amplification and sequencing using appropriately designed primers^25^. Sequence variants were detected via a comparison with reference sequences in GenBank (i.e., AF338659 for *cyp51A* and AFUB_020770 for *hmg1*).

### DNA extraction and whole-genome sequencing

Genomic DNA was extracted from mycelia derived from an overnight culture according to a published phenol-chloroform method^40^. Genomic DNA libraries of the *A. fumigatus* strains were constructed using the NEBNext Ultra DNA Library Prep Kit (New England BioLabs, Ipswich, MA). The 150-bp paired-end sequencing was performed using an Illumina HiSeq 4000 system (Illumina, San Diego, CA) by GENEWIZ (Saitama, Japan) or BGI (Shenzhen, China). An Illumina MiSeq system was used to generate 300-bp paired-end sequences of IFM 63345, IFM 63666, and IFM 63768.

### Single nucleotide polymorphism analysis

The raw genomic reads of all samples were screened for quality and trimmed using fastp v.0.20.1^47^. The filtered reads were aligned with the Af293 reference genome retrieved from AspGD (genome version: s03-m05-r04)^48^ using BWA-MEM v.0.7.17-r1188^49^. The mitochondrial genome was excluded for the analysis. SNPs were analyzed using GATK v.4.1.2.0^50^. According to the best practice workflow for ‘Germline short variant discovery’ of GATK^20,26,28,42^, the sorted BAM file for each sample was recalibrated using ‘BaseRecalibrator’ and known SNVs from FungiDB (release 56)^51^ as well as ‘ApplyBQSR’. Next, ‘HaplotypeCaller’ with ‘--sample-ploidy 1’ and the recalibrated BAM file for each sample were used to call short variants (SNPs and INDELs), after which ‘GenotypeGVCFs’ was used to combine the vcf files. Only SNPs were extracted from the joint-called variant file using ‘SelectVariants’. To eliminate false positives, ‘VariantFiltration’ was used with the following parameters as described in the GATK document: ‘QUAL < 30.0 || QD < 2.0 || FS > 60.0 || MQ < 40.0 || MQRankSum < −12.5 || ReadPosRankSum < −8.0 || SOR > 3.0’.

The pairwise comparison of the SNPs in IFM 62103 and IFM 62105-1 was performed using mpileup in SAMtools v.1.10^52^. The pileup vcf files were generated, and the consensus SNPs were excluded if they did not meet a minimum coverage of 10× or if the variant was present in <90% of the base calls by in-house scripts^8,53,54^.

### Phylogenetic analysis of whole-genome sequencing SNP data

The SNP sites with a minor allele frequency ≥5% and no missing data were filtered using VCFtools v.0.1.16 with the options ‘--maf 0.05 --max-missing 1’^55^. A phylogenetic tree was constructed using the multithreaded version of RAxML v.8.2.12^56^, the GTRCAT model, and 1,000 bootstrap replicates. The phylogenetic tree was visualized using the ggtree package^57^. Tajima’s *D* values were calculated using VCFtools with the option ‘--TajimaD 10000’. The numbers of pairwise SNPs between the strains of each cluster were calculated using snp-dists (https://github.com/tseemann/snp-dists).

### Population structure analysis

DAPC implemented in the adegenet package v.2.1.10^58^ was performed to assign the strains according to 68,816 loci. The vcfR package v.1.14.0^59^ was used for reading and parsing the vcf file. The function optim.a.score was iteratively used to determine the number of principal components used. Additionally, fastStructure v.1.0^60^ was used to estimate the population structure. The marginal likelihood values for each number of populations (K = 1–15) were calculated using 30 independent seeds. PCA was conducted using plink v.1.90^61^.

Overrepresented and underrepresented countries of each cluster were identified using Fisher’s exact test. The one-tailed Fisher’s exact *p*-value corresponding to overrepresentation and underrepresentation of a particular country have been calculated based on counts in 2 × 2 contingency tables. The *p*-values were corrected by the FDR method^62^.

*D*-statistic is a statistical test for admixture based on a four-cluster comparison^63^. The *D*-statistics were calculated using the f4 function implemented in the admixtools package v.2.0.0^64^.

Recombination analysis was performed using LDhat v.2.2a^65^. The interval program was used to estimate the recombination rates for each cluster, following generation of the lookup table by the lkgen program using “lk_n320_t0.01” for 320 sequences with theta = 0.01 (https://zenodo.org/records/3934350). The program was executed for 2 million iterations with sampling every 200 iterations after a 20,000-iteration burn-in period. The stat program was used for summarizing the results.

### Genome-wide association study and genomic selection on the basis of ridge regression

The 90,648 SNP sites that satisfied certain criteria (i.e., minor allele frequency ≥5% and ≤10% missing data) were used for the GWAS. MLM analysis was completed using TASSEL v.5^66^. Multidimensional scaling (MDS) and Kinship matrices were used as covariates to control the population structure. The ridge regression analysis was performed using the glmnet package v.4.1-8^67–69^. The log_2_-transformed MIC values were predicted according to genomic loci through a 5-fold cross validation using the function cv.glmnet. The SNPs were annotated using SnpEff v.5.1d^70^ and the annotated *A. fumigatus* Af293 reference genome.

### Determination of mating type idiomorphs

The mitochondrial genomes were assembled using GetOrganelle v.1.6.4^71^. To filter the mitochondrial reads, the reads were aligned with the mitochondrial genome using BWA. The mapped reads were filtered using SAMtools and SeqKit v.0.10.1^72^. The nuclear genomes were assembled using VelvetOptimiser v.2.2.6^73^. blastn v.2.5.0+^74^ was used for identifying MAT types, with MAT1-1 (AY89866.1) and MAT1-2 (Afu3g06170) serving as query sequences. The AFIS1704 genome size was estimated using GenomeScope^75^ with 21 k-mers.

### Reporting summary

Further information on research design is available in the Nature Portfolio Reporting Summary linked to this article.

### Supplementary information

Supplementary Information

Description of Additional Supplementary Files

Supplementary Data 1

Supplementary Data 2

Supplementary Data 3

Supplementary Data 4

Supplementary Data 5

Supplementary Data 6

Supplementary Data 7

Reporting Summary

## Data Availability

Raw reads have been deposited in the DDBJ BioProject database (BioProject accession number PRJDB16281). Source data underlying Fig. 1c can be found in Supplementary Data 4. The newick file underlying Fig. 2a can be found in Supplementary Data 6. Other source data underlying Figs. can be found in Supplementary Data 7.

## Code availability

Codes for analysis and visualization are available from the authors upon request.
