# Supplementary material for: Genomic diversity of the pathogenic fungus Aspergillus fumigatus in Japan reveals the complex genomic basis of azole resistance
Source: Commun Biol. 2024 Mar 14;7:274. doi: 10.1038/s42003-024-05902-6 (PMC10940670; doi:10.1038/s42003-024-05902-6)
Supplement: Supplementary file 1 — Supplementary Information [file 42003_2024_5902_MOESM1_ESM.pdf]

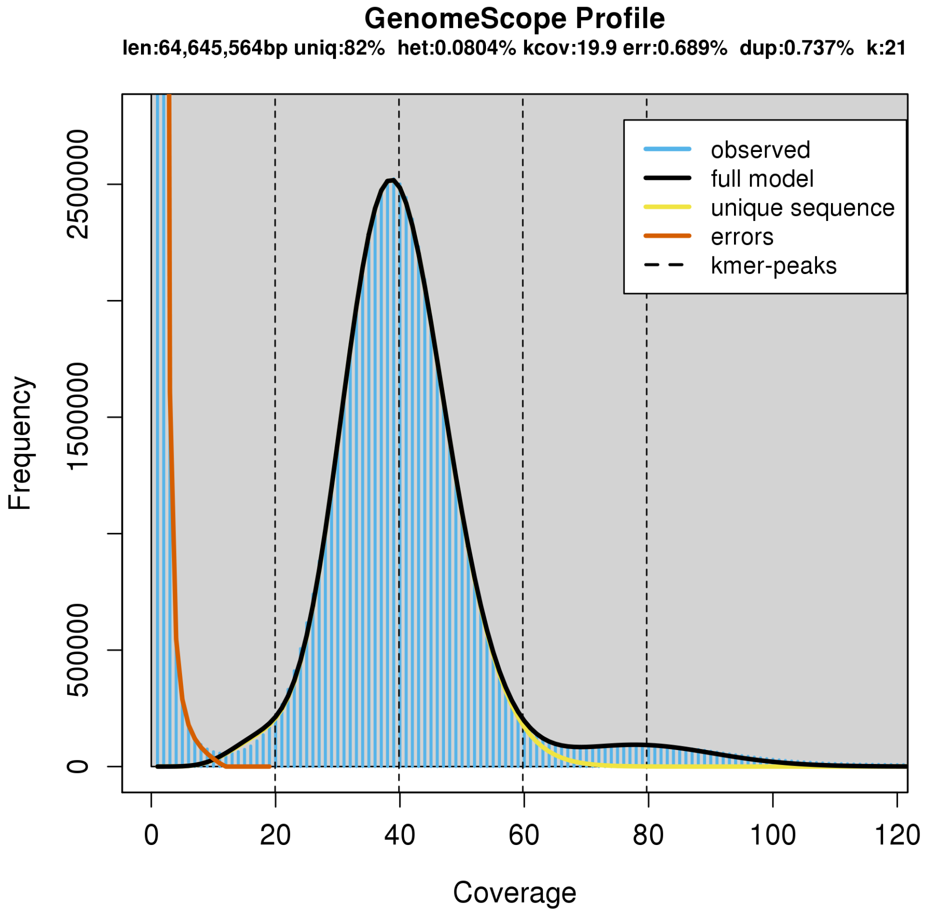

Supplementary Figure 1. Estimated size of the AFIS1704 genome. GenomeScope was used with 21 k-mers.

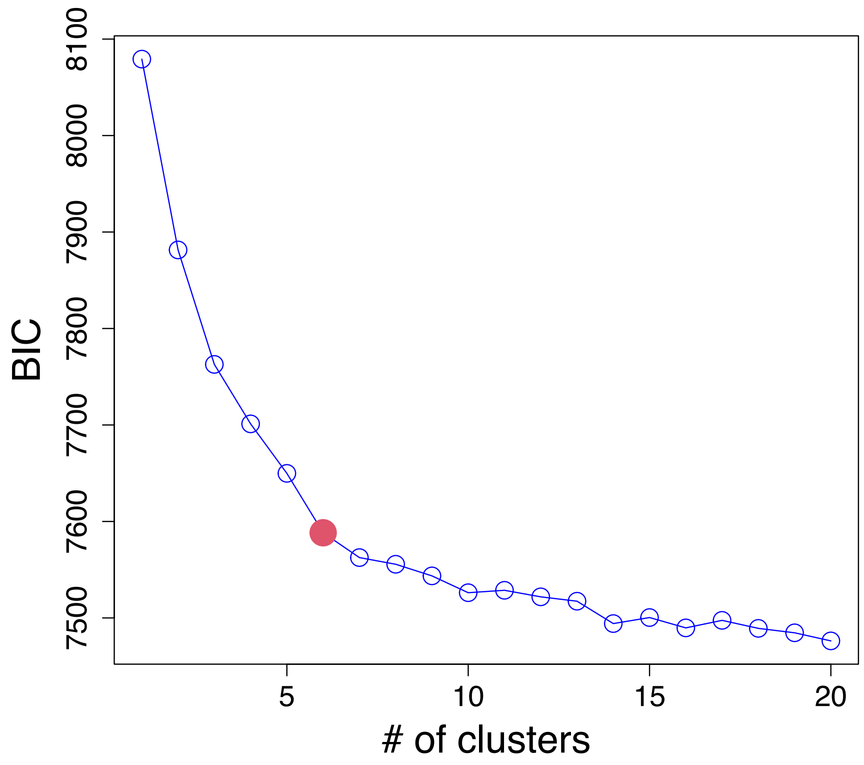

Supplementary Figure 2. Bayesian information criterion (BIC) values determined by DAPC. Red circle corresponds to K = 6. The x-axis and y-axis present numbers of clusters and BIC, respectively.

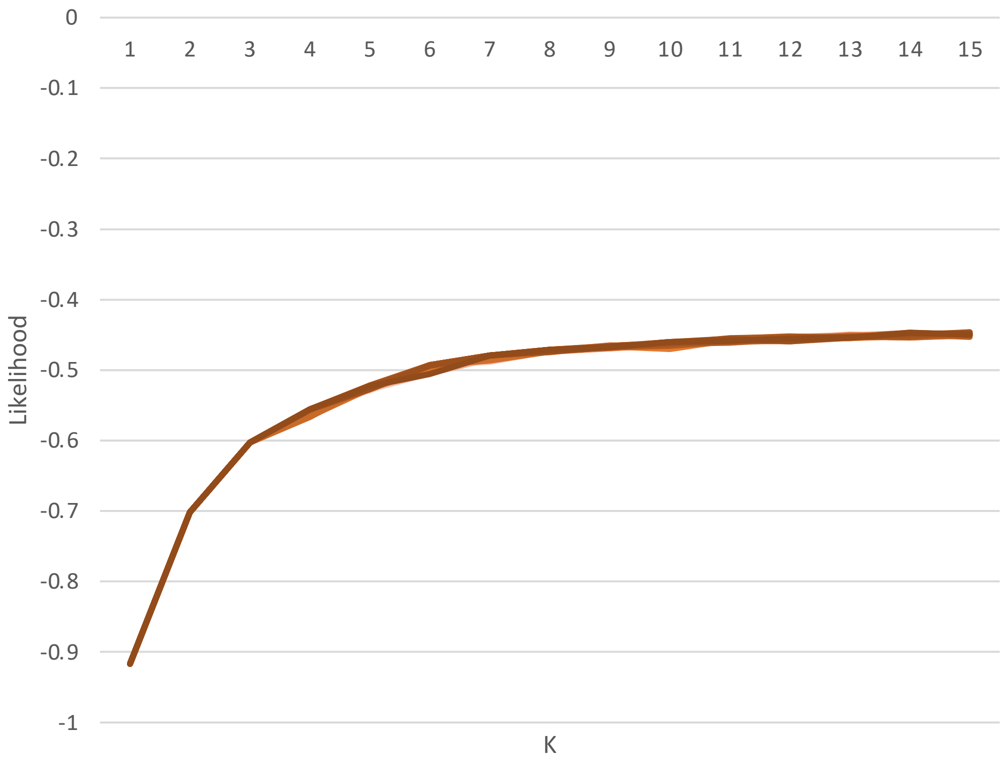

Supplementary Figure 3. Marginal likelihood values determined by fastStructure.

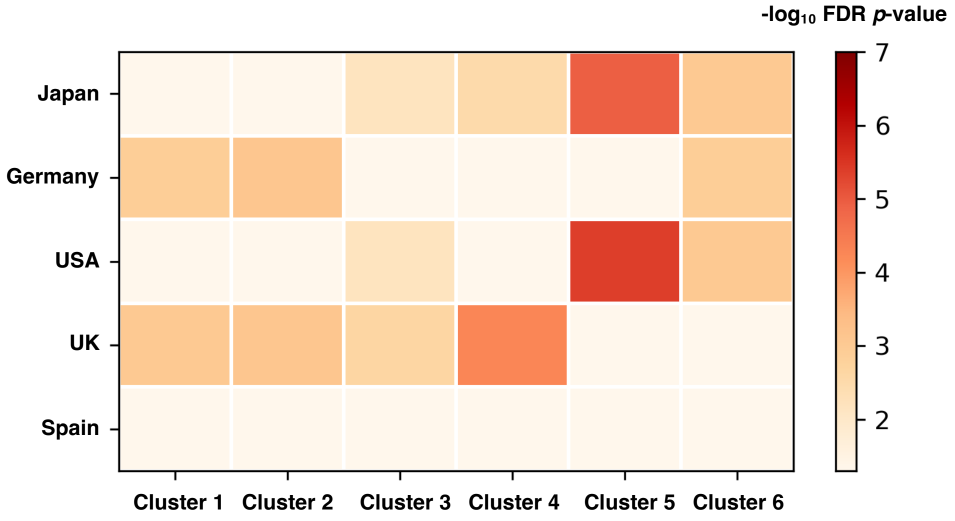

Supplementary Figure 4. Underrepresentation of geographic distributions of the strains for each cluster.

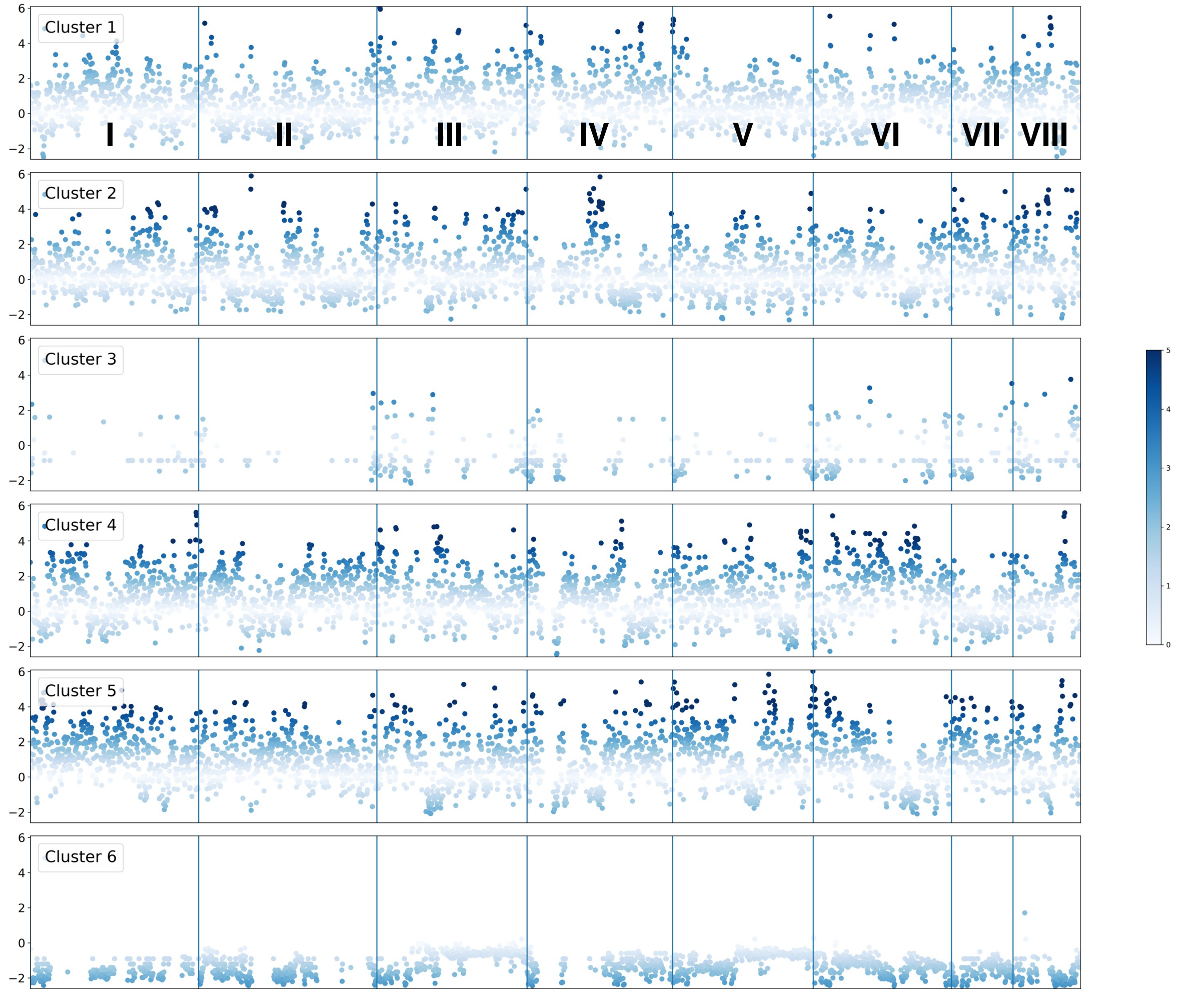

Supplementary Figure 5. Tajima's *D* values along the chromosome in each cluster.

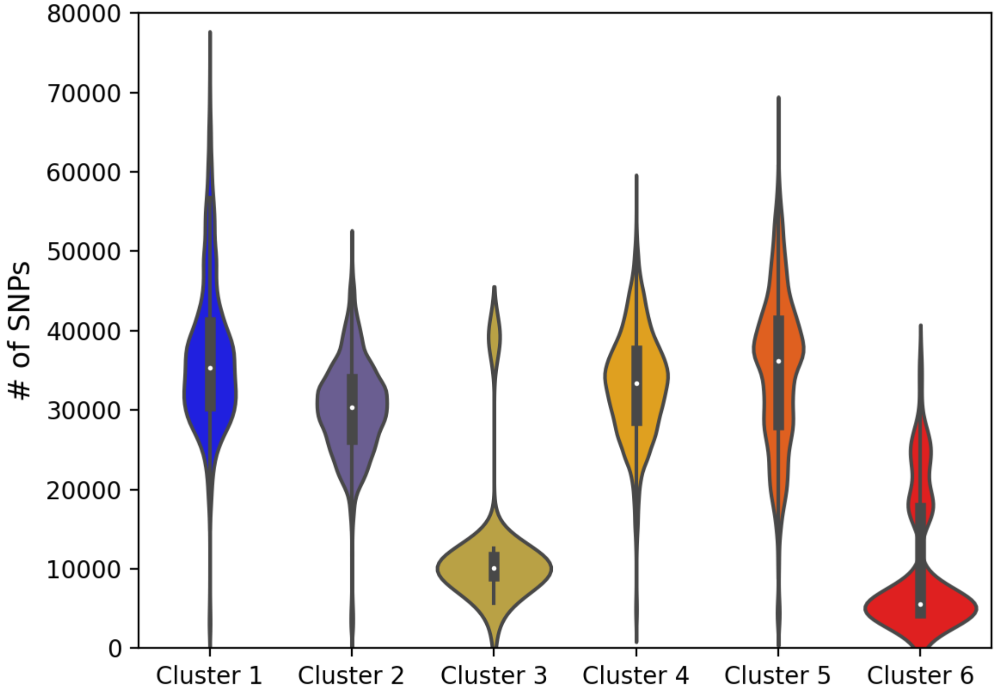

Supplementary Figure 6. Violin plots of the number of pairwise SNPs for each cluster. The numbers of pairwise comparison are 22,791 within Cluster 1, 17,020 within Cluster 2, 300 within Cluster 3, 28,920 within Cluster 4, 15,225 within Cluster 5 and 630 within Cluster 6. White dots and thick gray bar in the center represent the median and the interquartile range, respectively.

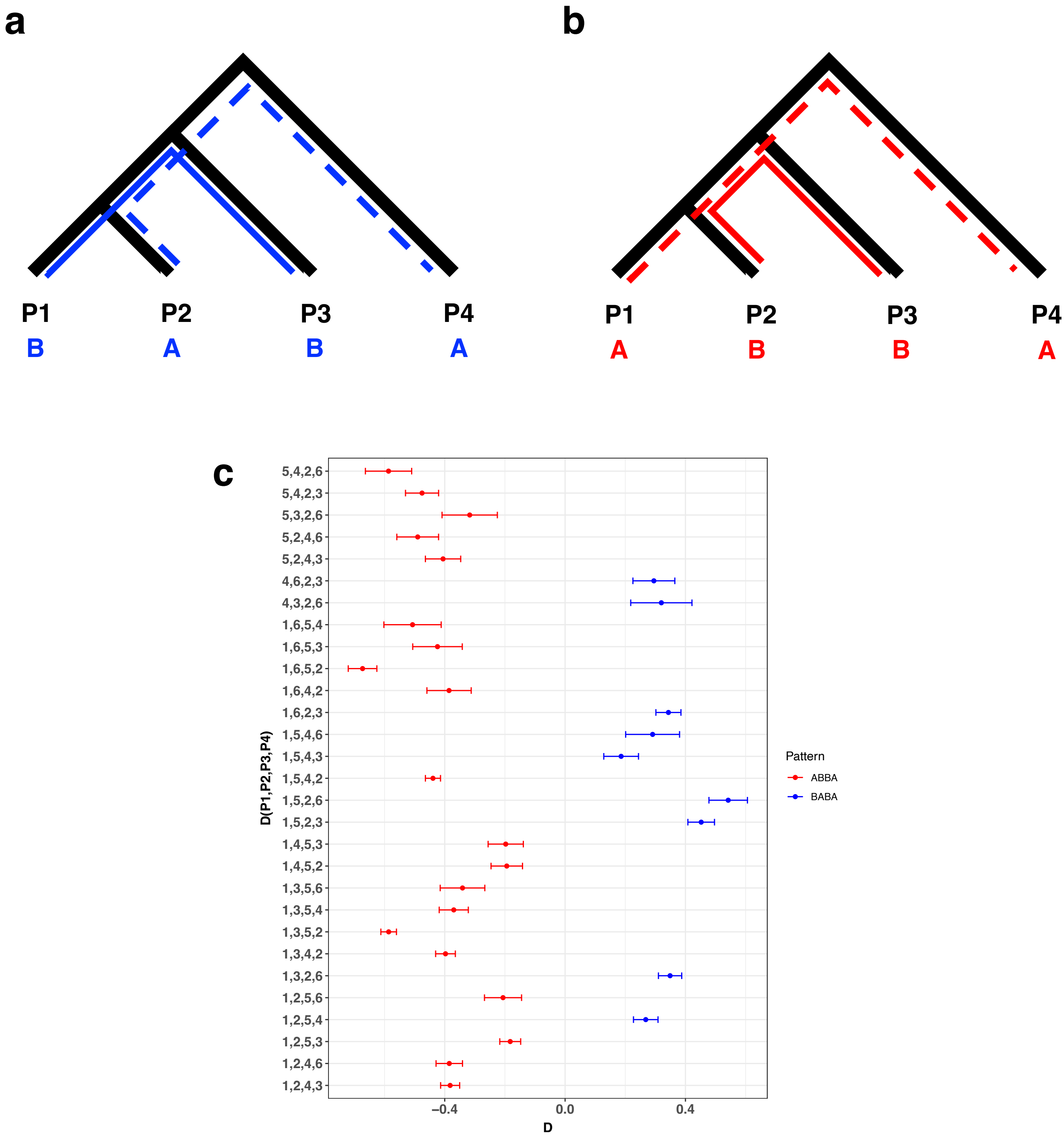

Supplementary Figure 7. 29 four-cluster comparisons with significant *D*-statistics. a The BABA-pattern. The positive *D*-statistic indicates an excess of BABA-pattern, that is, the gene flow occurred either between P1 and P3 or P2 and P4. **b** The ABBA-pattern. The negative *D*-statistic indicates an excess of ABBA-pattern, that is, the gene flow occurred either between P1 and P4 or P2 and P3. **c** 29 four-clusters comparisons. The x-axis and y-axis present *D*-statistics with standard error and D(P1, P2; P3, P4), respectively.

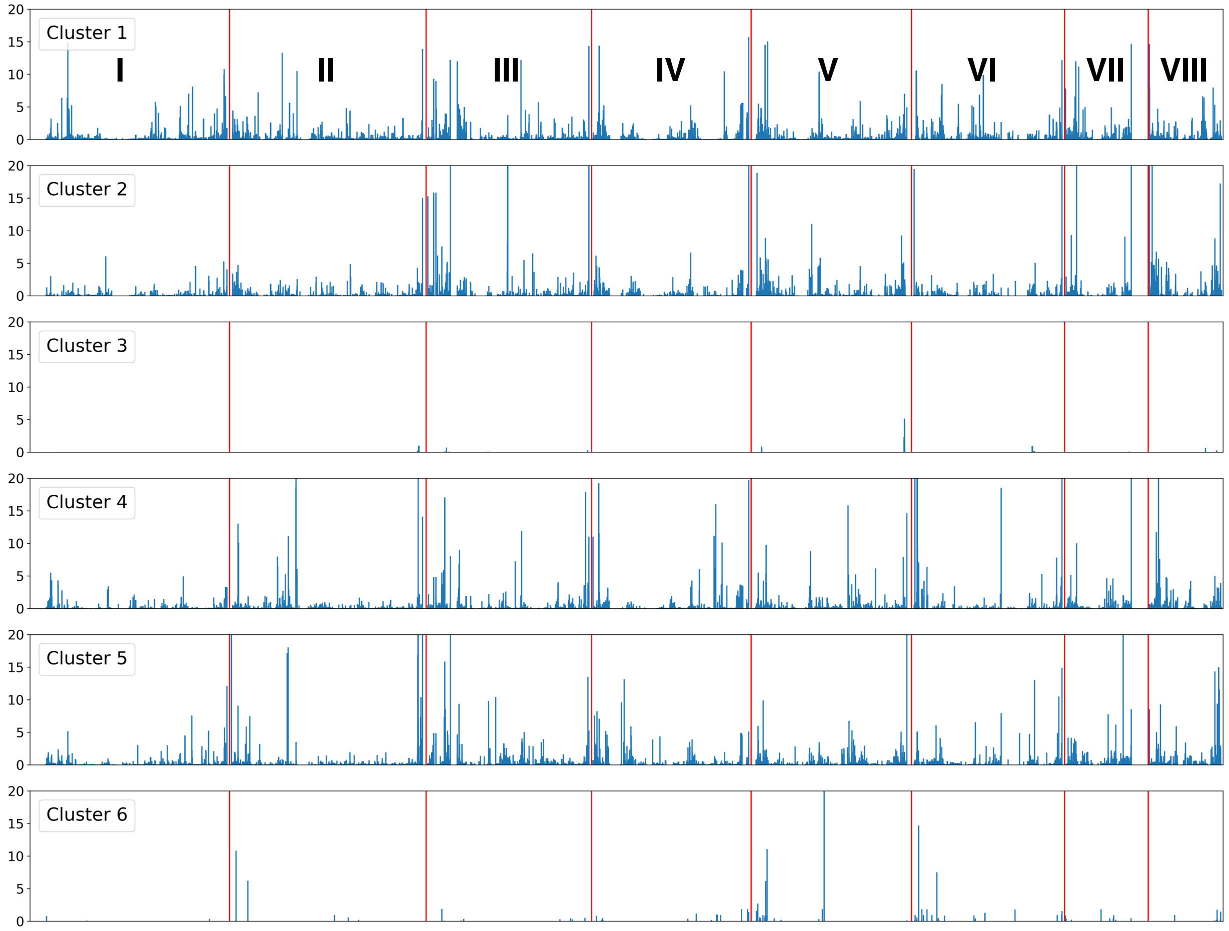

Supplementary Figure 8. Recombination analysis along the chromosome in each cluster. The x-axis and y-axis present chromosome loci and recombination rate $\rho(2N_e r)/bp$, respectively.

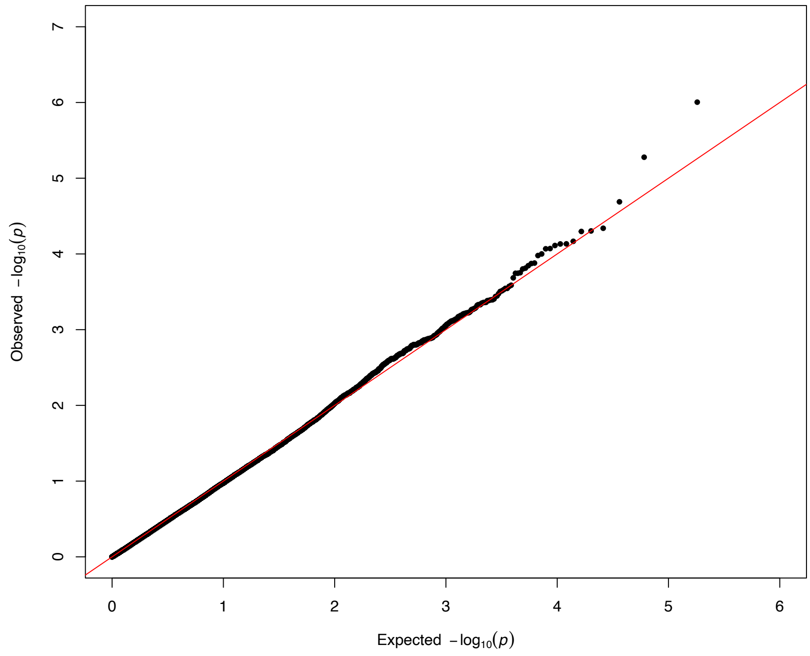

Supplementary Figure 9. Quantile–quantile plot.

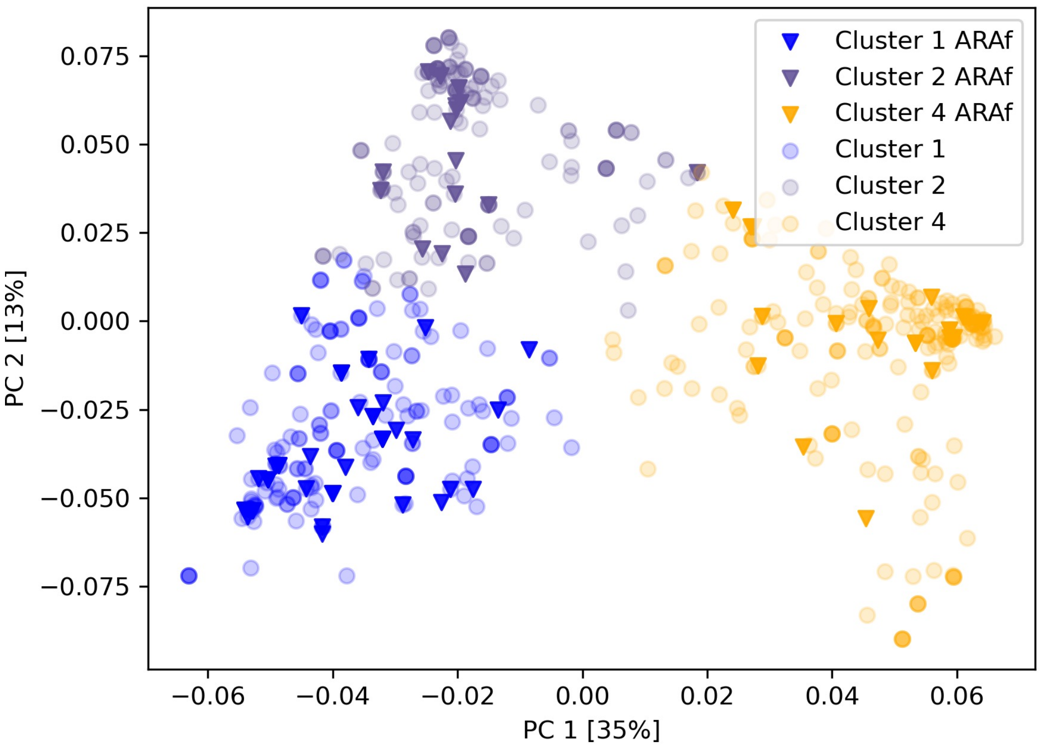

Supplementary Figure 10. Principal component analysis of 628 strains. The AR*Af* strains are indicated. The x-axis and y-axis present principal components (PCs) 1 and 2, respectively.

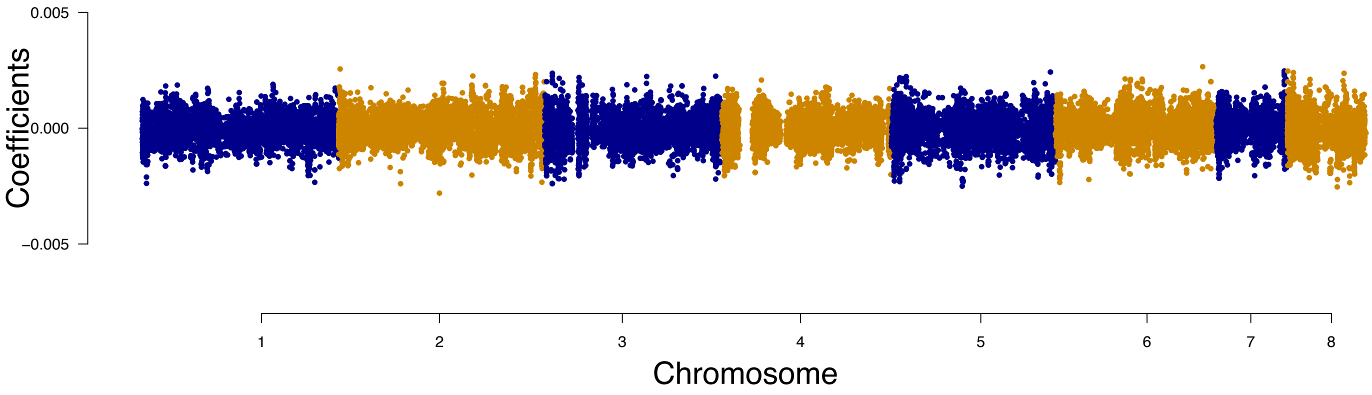

Supplementary Figure 11. The effectiveness of genomic loci determined by the ridge regression model. The x-axis and y-axis present 50,605 loci and the coefficients of genomic loci, respectively.

Supplementary Table 1. Overrepresentation and underrepresentation of geographic distributions for each cluster. FDR-corrected *p*-values are shown.

# Overrepresentation

Country	Cluster 1	Cluster 2	Cluster 3	Cluster 4	Cluster 5	Cluster 6
Japan	1.02E-02	4.52E-11	1.00E+00	1.00E+00	1.00E+00	1.00E+00
Germany	1.00E+00	1.00E+00	4.79E-02	2.96E-12	1.00E+00	1.00E+00
USA	5.85E-06	7.47E-02	1.00E+00	1.00E+00	1.00E+00	1.00E+00
UK	1.00E+00	1.00E+00	1.00E+00	1.00E+00	7.16E-15	6.60E-15
Spain	1.00E+00	1.00E+00	2.18E-12	1.00E+00	1.00E+00	1.00E+00

# Underrepresentation

Country	Cluster 1	Cluster 2	Cluster 3	Cluster 4	Cluster 5	Cluster 6
Japan	1.00E+00	1.00E+00	6.70E-03	3.00E-03	1.15E-05	8.74E-04
Germany	1.19E-03	7.20E-04	1.00E+00	1.00E+00	5.74E-01	1.19E-03
USA	1.00E+00	1.00E+00	6.70E-03	7.76E-01	4.14E-06	8.74E-04
UK	8.74E-04	7.20E-04	2.30E-03	5.22E-05	1.00E+00	1.00E+00
Spain	5.35E-01	5.18E-01	1.00E+00	8.13E-02	3.31E-01	5.28E-01

Supplementary Table 2. Average Tajima's *D* values per chromosome for strains within each cluster

Chr	Cluster 1	Cluster 2	Cluster 3	Cluster 4	Cluster 5	Cluster 6
1	0.69	0.48	-0.34	0.83	1.32	-1.60
2	0.29	0.36	-0.35	0.88	0.97	-1.51
3	0.95	0.67	-0.60	0.89	0.76	-0.89
4	0.94	0.27	-0.72	0.61	0.80	-1.36
5	0.51	0.33	-0.87	0.77	1.17	-1.03
6	0.53	0.47	-0.62	1.31	0.72	-1.38
7	0.54	0.93	-0.49	-0.07	1.24	-1.53
8	1.02	0.96	-0.27	0.73	0.92	-1.72

Supplementary Table 3. Summary of the numbers of pairwise SNPs within each cluster

	Cluster 1	Cluster 2	Cluster 3	Cluster 4	Cluster 5	Cluster 6
# of pairs	22,791	17,020	300	28,920	15,225	630
mean	36,707	30,117	12,019	33,158	34,975	10,314
std	9,472	6,325	8,277	6,903	9,942	8,304
min	1,587	1,874	971	2,575	3,217	4,039
25%	30,513	26,295	9,182	28,775	28,224	4,497
50%	35,339	30,327	10,157	33,377	36,233	5,548
75%	41,036	33,937	11,504	37,513	41,245	17,698
max	75,129	50,817	40,267	57,844	66,547	36,154

Supplementary Table 4. *D*-statistics based on four-cluster comparison. Among 45 four-cluster comparisons, 9 and 20 comparisons exhibited significant *D*-statistics with Z-score > 3 and Z-score < –3, respectively.

pop1 (A)	pop2 (B)	pop3 (C)	pop4 (D)	D-statistics	Standard error	Z	*p*-value	Number of SNPs
1	5	4	2	-0.44	0.03	-17.51	1.28E-68	68,816
1	4	5	2	-0.19	0.05	-3.71	2.10E-04	68,816
1	2	5	4	0.27	0.04	6.57	5.03E-11	68,816
1	5	4	6	0.29	0.09	3.25	1.17E-03	68,816
1	4	5	6	-0.25	0.12	-2.20	2.80E-02	68,816
1	6	5	4	-0.51	0.10	-5.32	1.01E-07	68,816
1	5	4	3	0.19	0.06	3.23	1.24E-03	68,816
1	4	5	3	-0.20	0.06	-3.37	7.45E-04	68,816
1	3	5	4	-0.37	0.05	-7.65	1.95E-14	68,816
1	5	2	6	0.54	0.06	8.47	2.41E-17	68,816
1	2	5	6	-0.21	0.06	-3.35	8.16E-04	68,816
1	6	5	2	-0.67	0.05	-14.16	1.58E-45	68,816
1	5	2	3	0.45	0.04	10.23	1.53E-24	68,816
1	2	5	3	-0.18	0.03	-5.25	1.55E-07	68,816
1	3	5	2	-0.59	0.03	-22.61	3.59E-113	68,816
1	5	6	3	-0.10	0.10	-0.94	3.48E-01	68,816
1	6	5	3	-0.42	0.08	-5.15	2.63E-07	68,816
1	3	5	6	-0.34	0.07	-4.60	4.26E-06	68,816
1	4	2	6	0.00	0.11	0.01	9.93E-01	68,816
1	2	4	6	-0.38	0.04	-8.76	1.99E-18	68,816
1	6	4	2	-0.39	0.07	-5.24	1.60E-07	68,816
1	4	2	3	0.02	0.06	0.30	7.66E-01	68,816
1	2	4	3	-0.38	0.03	-12.06	1.81E-33	68,816
1	3	4	2	-0.40	0.03	-12.13	7.20E-34	68,816
1	4	6	3	0.02	0.10	0.17	8.61E-01	68,816
1	6	4	3	0.08	0.10	0.78	4.32E-01	68,816
1	3	4	6	0.06	0.07	0.94	3.45E-01	68,816
1	2	6	3	-0.01	0.05	-0.12	9.02E-01	68,816
1	6	2	3	0.34	0.04	8.25	1.65E-16	68,816
1	3	2	6	0.35	0.04	9.02	1.92E-19	68,816
5	4	2	6	-0.59	0.08	-7.63	2.26E-14	68,816
5	2	4	6	-0.49	0.07	-7.07	1.59E-12	68,816
5	6	4	2	0.14	0.10	1.41	1.58E-01	68,816
5	4	2	3	-0.48	0.06	-8.64	5.44E-18	68,816
5	2	4	3	-0.41	0.06	-6.92	4.59E-12	68,816
5	3	4	2	0.09	0.05	1.59	1.13E-01	68,816
5	4	6	3	0.11	0.10	1.11	2.68E-01	68,816
5	6	4	3	-0.14	0.10	-1.41	1.59E-01	68,816
5	3	4	6	-0.25	0.10	-2.61	9.14E-03	68,816
5	2	6	3	0.10	0.10	0.99	3.22E-01	68,816
5	6	2	3	-0.23	0.08	-2.66	7.80E-03	68,816
5	3	2	6	-0.32	0.09	-3.45	5.62E-04	68,816
4	2	6	3	-0.03	0.10	-0.28	7.80E-01	68,816
4	6	2	3	0.30	0.07	4.24	2.27E-05	68,816
4	3	2	6	0.32	0.10	3.14	1.67E-03	68,816
