# Supplementary material for: Genomic diversity of the pathogenic fungus Aspergillus fumigatus in Japan reveals the complex genomic basis of azole resistance
Source: Commun Biol. 2024 Mar 14;7:274. doi: 10.1038/s42003-024-05902-6 (PMC10940670; doi:10.1038/s42003-024-05902-6)
Supplement: Supplementary file 2 — Description of Additional Supplementary Files [file 42003_2024_5902_MOESM2_ESM.pdf]

Description of Additional Supplementary Files

File name: Supplementary Data 1

Description: Details regarding the 173 strains used in this study

File name: Supplementary Data 2

Description: Information regarding the SNPs between IFM 62103 and IFM 62105-1

File name: Supplementary Data 3

Description: Cluster assignment by DAPC with posterior probabilities

File name: Supplementary Data 4

Description: Probabilities for the assignments determined by fastStructure

File name: Supplementary Data 5

Description: Details regarding the 876 strains

File name: Supplementary Data 6

Description: Newick file for phylogeny

File name: Supplementary Data 7

Description: The source data behind the graphs in the paper
